# Temporal trends and regional clusters of tuberculosis-HIV coinfection outcomes in Brazil

**DOI:** 10.11606/s1518-8787.2025059006736

**Published:** 2025-11-05

**Authors:** Lucas Vinícius de Lima, Gabriel Pavinati, Yenny Lorena Valdivia-Rojas, José Nildo de Barros Silva, Ana Paula Sayuri Sato, Fredi Alexander Diaz-Quijano, Victor Santana Santos, Gabriela Tavares Magnabosco

**Affiliations:** I Universidade Estadual de Maringá Centro de Ciências da Saúde Programa de Pós-Graduação em Enfermagem Maringá PR Brasil Universidade Estadual de Maringá. Centro de Ciências da Saúde. Programa de Pós-Graduação em Enfermagem. Maringá, PR, Brasil; II Prefeitura Municipal de Maringá Secretaria Municipal de Saúde Gerência de Planejamento Maringá PR Brasil Prefeitura Municipal de Maringá. Secretaria Municipal de Saúde. Gerência de Planejamento. Maringá, PR, Brasil; III Universidad de Antofagasta Departamento de Enfermería Unidad Académica de Enfermería Antofagasta AN Chile Universidad de Antofagasta. Departamento de Enfermería. Unidad Académica de Enfermería. Antofagasta, AN, Chile; IV Universidade de São Paulo Escola de Enfermagem de Ribeirão Preto Programa de Pós-Graduação em Enfermagem em Saúde Pública Ribeirão Preto SP Brasil Universidade de São Paulo. Escola de Enfermagem de Ribeirão Preto. Programa de Pós-Graduação em Enfermagem em Saúde Pública. Ribeirão Preto, SP, Brasil; V Universidade de São Paulo Faculdade de Saúde Pública Departamento de Epidemiologia São Paulo SP Brasil Universidade de São Paulo. Faculdade de Saúde Pública. Departamento de Epidemiologia. São Paulo, SP, Brasil; VI Universidade Federal de Sergipe Departamento de Medicina Programa de Pós-Graduação em Ciências Aplicadas à Saúde Lagarto SE Brasil Universidade Federal de Sergipe. Departamento de Medicina. Programa de Pós-Graduação em Ciências Aplicadas à Saúde. Lagarto, SE, Brasil

**Keywords:** HIV, Tuberculosis, Ecological Studies, Time Series Studies, Cluster Analysis

## Abstract

**OBJECTIVE:**

To investigate the temporal trends and regional clusters of tuberculosis treatment outcomes among people with HIV in Brazil, as well as their relationship with socioeconomic and programmatic indicators.

**METHODS:**

Ecological study with data from people living with HIV who initiated and completed tuberculosis treatment from 2015 to 2021 in Brazil. We described the semiannual trends of cure, treatment interruption, and death rates in Brazilian states using joinpoint regression models. Cluster analyses stratified by three population sizes were conducted using the k-means method to identify clusters in 510 immediate geographic regions. Socioeconomic and programmatic indicators related to treatment interruption and death were included in multivariate negative binomial regression models.

**RESULTS:**

A total of 54,362 tuberculosis treatments in people with HIV were analyzed, with cure rates of 55.51%, interruption rates of 23.33%, and death rates of 21.16% for the period. Nationally, cure rates remained stable, while treatment interruption increased by 2.54% per semester (ranging from 1.59% to 3.70%) and death increased by 9.31% (ranging from 7.41% to 17.24%). The states of Ceará and Amapá showed the worst trends for treatment interruption and death. Regions with greater income inequality, higher percentages of workers with only primary education, higher household density, and broader private health coverage had higher rates of treatment interruption and death. Conversely, regions with higher expected years of schooling and hospitalizations for primary care-sensitive conditions had lower probabilities of these outcomes.

**CONCLUSION:**

Nationally, despite stable cure rates, tuberculosis treatment interruption and death among people with HIV increased. Regional disparities in the relationship between socioeconomic and programmatic indicators and outcomes suggest inequities in access to and adherence to tuberculosis treatment across Brazil.

## INTRODUCTION

Tuberculosis coinfection among people living with human immunodeficiency virus (HIV) represents one of the greatest public health challenges. Tuberculosis, historically associated with socioeconomic vulnerability, becomes even more complex with HIV due to the immunosuppression involved^1^. According to the World Health Organization (WHO)^2^, an estimated 10.8 million people developed tuberculosis worldwide in 2023, of whom approximately 6.1% were HIV-positive^2^.

In Brazil, 80,000 new tuberculosis cases were reported in 2023; of these, an estimated 9.3% involved HIV coinfection^4^. From 2010 to 2021, there was an annual decrease of −4.3% (ranging from −5.1% to −3.7%) in the incidence of coinfection^5^. The country still faces a significant burden of these conditions and has implemented several policies to control tuberculosis in people with HIV. However, barriers related to treatment completion hinder the national response^6,7^.

Addressing tuberculosis-HIV coinfection is a priority on the global agenda, directly addressed in the United Nations Sustainable Development Goals, which aim to eliminate tuberculosis and reduce the impact of HIV by 2030^8^. To achieve this target, countries must strengthen their health systems via adequate funding^8^, ensuring universal access to early diagnosis and timely treatment, especially for vulnerable populations.

In Brazil, cure rates reached around 50.6% in 2020, with improvements among those receiving antiretroviral therapy and with CD4+ lymphocyte counts >350 cells/mm^3^; treatment interruption and death accounted for 16.9% and 21.8% of cases, respectively^6^. Studies indicate that drug interactions and adverse effects, financial constraints, weaknesses in health services, and social vulnerabilities associated with these infections may hinder completion of tuberculosis therapy among people with HIV^7,9^.

The 2030 agenda requires an approach that considers these barriers in an intersectoral manner to optimize therapeutic responses to coinfection in Brazil. To this end, it is essential to identify patterns in treatment outcomes, revealing areas that require strategic actions. In this context, this study investigated the temporal trends and regional clusters of tuberculosis treatment outcomes among people with HIV in Brazil, considering their relationship with socioeconomic and programmatic indicators.

## METHODS

This was an ecological study using surveillance data on tuberculosis treatment in people with HIV in Brazil, obtained from health information systems. Although aggregated data were used, granted by the Brazilian Ministry of Health via a request submitted on the Fala.BR Platform (protocol no. 25072.039887/2022-27), approval was obtained from the Research Ethics Committee (opinion no. 6.914.233/2024), as the study integrates an academic multimethod research project, in accordance with Resolution no. 674/2022 of the Brazilian National Health Council.

The study covered Brazil, which has an approximate area of 8.5 million km^2^ and a population of about 203 million people^12^. Brazil—the largest country in Latin America—comprises 5,570 municipalities and districts, which are administratively organized into 27 federative units. At a lower level of aggregation, they form 510 immediate geographic regions (IGRs), defined by the connection of cities via dependency relations and population mobility to meet immediate needs such as health, education, and employment.

The data were obtained via probabilistic linkage, as described by the Brazilian Ministry of Health^6^, between the following systems: the National Notifiable Diseases Information System (SINAN), the Mortality Information System (SIM), the Antiretroviral Drug Dispensing System (SICLOM), and the HIV Laboratory Examination System (SISCEL). In total, three linkages were performed: (i) among HIV databases; (ii) among tuberculosis databases; and (iii) between the outputs of these stages^6^.

The study population consisted of people with HIV who initiated tuberculosis treatment between January 2015 and June 2021 and completed it by December 2021. Included were those with the variables “HIV” or “AIDS” coded as “yes” in the SINAN-tuberculosis database, or those without this coding but with records in SINAN-HIV, SIM-HIV, SICLOM, or SISCEL^6^. New, relapse, or retreatment cases of tuberculosis with “treatment outcome” not filled in or filled in with operational outcomes (9%) were excluded.

The main variable was extracted from “treatment outcome” in SINAN, defined as follows: “cure,” corresponding to completion/discharge from tuberculosis treatment; “primary abandonment” and “abandonment” (i.e., “treatment interruption”), representing treatment discontinuation before and after 30 days from initiation, respectively, with absence for 30 consecutive days; and “death from tuberculosis” and “death from other causes” (i.e., “death”), covering deaths with tuberculosis listed as the underlying or associated cause in SIM.

Secondary variables were extracted from the Atlas of Human Development in Brazil (Atlas Brasil)^13^, an online platform providing data on over 300 indicators reflecting living conditions in the country in the social, economic, political, and environmental dimensions. Based on previous studies and hypotheses, a set of socioeconomic and programmatic indicators of Brazilian municipalities—also referred to as contextual indicators in this study—was selected and is presented in Box.

**Box:** Description of socioeconomic and programmatic (contextual) indicators, measured at the municipal level, selected for this study, Brazil, 2024.

Code	Description	Source
GINI	GINI index: measures the degree of inequality in the distribution of individuals by per capita household income; its value ranges from 0.00 (no inequality, i.e., all individuals have the same per capita household income) to 1.00 (maximum inequality, i.e., only one individual holds all income).	IBGE (2010)
PER_OCUPEF	Percentage of employed individuals with completed primary education: ratio between the number of employed persons aged 18 years or older who have completed primary education and the total number of employed persons in this age group, multiplied by 100.	IBGE (2010)
PER_DOMIC	Percentage of the population living in households with a bathroom and piped water: ratio between the population living in permanent private households with piped water in at least one room and an exclusive bathroom, and the total resident population in permanent private households, multiplied by 100.	IBGE (2010)
PER_DENSI	Percentage of the population living in households with density greater than two persons per bedroom: ratio between the population living in permanent private households with density greater than two persons per bedroom and the total resident population in permanent private households, multiplied by 100.	IBGE (2010)
ESP_VIDA	Life expectancy at birth: average number of years a person is expected to live from birth, assuming the age-specific mortality levels and patterns prevailing in the census year remain constant throughout life.	IBGE (2010)
IDHM	Municipal human development index: geometric mean of the indices related to income, education, and longevity, with equal weights; ranges from 0.00 to 1.00, from low to very high development, respectively.	IBGE (2010)
RAZ_DEPEN	Dependency ratio: ratio between the number of individuals aged 14 years or younger and 65 years or older (dependent population) and the number of individuals aged 15 to 64 years (potentially active population), multiplied by 100.	IBGE (2010)
RENDA	Per capita income: ratio between the total household income of all residents in permanent private households and the total number of such individuals.	IBGE (2010)
PER_VULPOB	Percentage vulnerable to poverty: proportion of individuals with a per capita household income less than or equal to 255.00 BRL per month, equivalent to half the minimum wage at the time.	IBGE (2010)
PER_POB	Percentage of poor individuals: proportion of individuals with a per capita household income less than or equal to 140.00 BRL per month.	IBGE (2010)
EXP_ESTU	Expected years of schooling at age 18: average number of years of schooling a generation of children entering school is expected to complete by age 18, assuming current patterns remain constant over their school life.	IBGE (2010)
PER_25EF	Percentage of individuals aged 25 years or older with completed primary education: ratio between the population aged 25 years or older who completed primary education in any form and the total population in this age group, multiplied by 100.	IBGE (2010)
TX_ENVEL	Aging rate: ratio between the population aged 65 years or older and the total population, multiplied by 100.	IBGE (2010)
PER_SAUSUPL	Percentage of the population covered by private health insurance: percentage share of the population covered by private health insurance plans relative to the total population.	SIB (2017)
PER_ICSAP	Percentage of hospitalizations for ambulatory care-sensitive conditions: percentage share of hospitalizations for ambulatory care-sensitive conditions in total hospital admissions.	SIH/SUS (2017)
PER_CADPBF	Percentage of individuals registered in the Unified Registry (CadÚnico) who receive *Bolsa Família* benefits: percentage of individuals in the CadÚnico whose family receives Bolsa Família, relative to the total registered population.	CadÚnico (2017)
PER_IDRSAI	Percentage of hospitalizations due to diseases related to inadequate sanitation: percentage share of hospital admissions due to sanitation-related diseases in total hospital admissions.	SIH/SUS (2017)
TX_AIDS	AIDS incidence rate: ratio between the number of new AIDS cases (ICD-10 codes B20 to B24) recorded in a given year and the total population, multiplied by 100,000.	SINAN (2017)
TX_MORT	Crude mortality rate: total number of deaths in the population, multiplied by 100,000.	SIM (2017)
TX_ANALF	Illiteracy rate (18 years or older): ratio between the population aged 18 years or older who cannot read or write a simple note and the total population in this age group, multiplied by 100.	IBGE (2010)
TX_MORTDCNT	Mortality rate from noncommunicable diseases: number of deaths from noncommunicable diseases in the total population, multiplied by 100,000.	SIM (2017)
TX_MORTINF	Infant mortality rate: ratio between the number of children not expected to survive their first year of life and the number of live births, multiplied by 1,000.	IBGE (2010)

IBGE: Brazilian Institute of Geography and Statistics; SIB: Health Insurance Beneficiaries Information System; CadÚnico: Unified Registry; SIH/SUS: Hospital Information System of the Unified Health System; ICD-10: International Classification of Diseases – 10th revision; SINAN: National Notifiable Diseases Information System; SIM: Mortality Information System.

Initially, cure, treatment interruption, and death rates were estimated for each state as the proportion between the number of people with the outcome of interest and the total number of reported cases in that location; the result was multiplied by 100. Due to the temporal structure of the dataset, semiannual rates were used based on the treatment completion date, thereby increasing the number of points analyzed. In total, 13 semiannual points were obtained for trend estimation.

Trend assessment was performed using joinpoint regression models, suitable for time series analysis as they account for autocorrelation^14^. The rates of each outcome, transformed using the natural logarithm for better interpretation and comparison, were considered as the dependent variable, and the semester calendar of the period as the independent variable. The log-linear models were adjusted for the standard errors of the rates and for first-order autocorrelation, when present.

For the final models of each series, semiannual percentage changes (SPC), referring to the relative change in semiannual rate values throughout the study period, were estimated using the empirical quantile method, along with their 95% confidence intervals (95%CI). Semiannual variations whose 95%CI did not include zero (0.00) were considered significant, indicating increasing or decreasing trends. Analyses were performed using the Joinpoint Regression Program® (version 5.0.2).

The variables listed in Box were described using means and standard deviations (SD). To identify factors associated with treatment interruption and death from tuberculosis in people with HIV, regression models with a negative binomial distribution were applied, given evidence of data overdispersion^15^. In these models, event counts were treated as the dependent variable, contextual indicators as the independent variables, and the total number of cases in the corresponding IGR as the offset.

For regression models, independent variables were aggregated into the 510 IGRs via population-weighted means, using the 2022 census municipal population as the weighting factor. These variables were standardized by subtracting the mean and dividing by the SD to facilitate comparison of effect sizes. Measures of association were expressed as rate ratios (RR), considered significant when their 95%CI differed from 1.00 (the null value).

Each independent variable was first evaluated in bivariate analyses for each negative outcome. To obtain parsimonious multiple models, collinear variables were avoided. When correlation was identified by Spearman’s coefficient, the variable with the strongest association with the outcome in the bivariate analysis was selected for the multiple model. A backward stepwise selection method was used, retaining variables with p-value < 0.05 in the final models.

Using the variables included in the final model, a non-hierarchical cluster analysis was performed with the k-means method, which partitions the dataset into k groups by minimizing the sum of squared distances between data points and their respective centroids^16^. In this study, the k value was defined by the elbow method. Given the wide variation in the number of inhabitants among IGRs, clustering was stratified by population size: < 100,000 inhabitants (stratum I), 100,000–300,000 inhabitants (stratum II), and > 300,000 inhabitants (stratum III).

For each population stratum, the assumption was that data within each cluster would be more similar to one another than to data in other clusters of the same stratum, based on the distance between points and centroids. Additionally, a choropleth map was created using QGIS® software (version 2.36.3) to illustrate the profile of the clusters generated in each stratum. The remaining analyses were conducted using SPSS Statistics® software (version 25.0).

## RESULTS

A total of 54,362 cases of tuberculosis treatment in people with HIV in Brazil were analyzed. The cure rate was 55.51%, while treatment interruption and death accounted for 23.33% and 21.16%, respectively, over the study period. In the trend analysis, a stationary series was observed for treatment cure rates nationwide, with a semiannual variation of −1.02% (95%CI: −4.33% to 2.41%); treatment interruption and death showed semiannual increases of 2.54% (95%CI: 1.59% to 3.70%) and 9.31% (95%CI: 7.41% to 17.24%), respectively.

A semiannual decline in cure was observed in Ceará (SPC = −3.24%; 95%CI: −5.83% to −0.82%) and in the Federal District (SPC = −2.39%; 95%CI: −4.13% to −1.35%). Regarding treatment interruption, nine states showed increases, with the highest in Amapá (SPC = 21.35%; 95%CI: 9.99% to 33.72%) and Ceará (SPC = 5.09%; 95%CI: 2.18% to 8.94%). For death, only Espírito Santo showed a decline of −14.68% (95%CI: −21.44% to −6.94%), whereas five states showed increases, most notably Ceará (SPC = 13.63%; 95%CI: 10.22% to 26.34%) (Table 1).

**Table 1:** Semiannual trends in cure, treatment interruption, and death rates from tuberculosis in people with HIV, by federal unit, Brazil, 2015–2021.

Location	Cure	Interruption	Death
	SPC (95%CI)	SPC (95%CI)	SPC (95%CI)
North			
Rondônia	−1.56 (−4.06 to 0.50)	4.84 (1.67 to 8.26)	−3.64 (−12.13 to 4.57)
Acre	0.04 (−0.27 to 0.46)	−0.00 (−0.29 to 0.12)	−5.46 (−15.05 to 5.36)
Amazonas	0.30 (−3.09 to 3.84)	2.97 (−0.01 to 5.46)	9.00 (−6.80 to 26.50)
Roraima	0.41 (−2.51 to 2.77)	−2.95 (−16.52 to 16.40)	3.52 (−1.13 to 9.02)
Pará	−1.03 (−3.22 to 1.48)	4.32 (2.09 to 6.84)	9.09 (2.82 to 19.22)
Amapá	−1.94 (−5.01 to 0.94)	21.35 (9.99 to 33.72)	1.18 (−5.81 to 10.09)
Tocantins	2.74 (−3.76 to 12.39)	3.51 (−9.12 to 17.56)	4.35 (−13.10 to 15.64)
Northeast			
Maranhão	0.13 (−2.03 to 2.70)	0.35 (−2.44 to 3.29)	3.17 (−1.49 to 8.65)
Piauí	−2.04 (−5.12 to 0.58)	−1.87 (−7.83 to 3.53)	11.11 (7.09 to 16.83)
Ceará	−3.24 (−5.83 to −0.82)	5.09 (2.18 to 8.94)	13.63 (10.22 to 26.34)
Rio Grande do Norte	−1.03 (−5.52 to 4.05)	−0.97 (−11.48 to 11.20)	3.55 (−3.45 to 11.40)
Paraíba	−2.44 (−7.79 to 2.81)	3.88 (−4.22 to 14.21)	2.44 (−7.19 to 15.12)
Pernambuco	−0.58 (−3.29 to 2.31)	3.58 (1.12 to 5.97)	4.70 (−1.73 to 12.85)
Alagoas	−2.23 (−7.68 to 2.33)	3.68 (−0.79 to 8.92)	5.76 (−3.43 to 16.36)
Sergipe	0.22 (−1.89 to 2.32)	1.93 (−2.09 to 7.01)	−1.15 (−10.30 to 9.12)
Bahia	−0.25 (−2.00 to 1.52)	−0.43 (−3.46 to 2.36)	2.99 (−1.96 to 7.87)
Southeast			
Minas Gerais	1.38 (−1.15 to 3.89)	−1.67 (−4.35 to 0.61)	1.47 (−4.52 to 6.74)
Espírito Santo	−10.20 (−26.26 to 7.44)	−12.43 (−21.52 to 0.50)	−14.68 (−21.44 to −6.94)
Rio de Janeiro	−0.35 (−2.31 to 1.68)	2.41 (0.81 to 4.28)	2.81 (−1.16 to 6.83)
São Paulo	−0.65 (−1.89 to 0.51)	2.72 (1.72 to 3.73)	0.65 (−3.55 to 4.86)
South			
Paraná	−0.01 (−3.49 to 3.37)	3.20 (−3.39 to 9.93)	5.05 (−3.84 to 12.78)
Santa Catarina	−0.24 (−2.70 to 2.10)	−0.33 (−6.87 to 6.07)	9.22 (0.67 to 19.91)
Rio Grande do Sul	−0.26 (−3.74 to 3.33)	2.23 (0.07 to 4.44)	3.96 (−0.74 to 9.08)
Central-West			
Mato Grosso	2.28 (−1.42 to 6.38)	−1.57 (−5.16 to 1.51)	4.56 (−7.30 to 16.07)
Mato Grosso do Sul	−0.56 (−5.30 to 5.48)	1.17 (−1.97 to 4.74)	5.22 (0.07 to 13.55)
Goiás	1.34 (−3.77 to 6.33)	0.17 (−6.24 to 5.83)	0.84 (−4.66 to 5.50)
Federal District	−2.39 (−4.13 to −1.35)	5.70 (2.02 to 9.56)	4.37 (−3.23 to 12.33)

SPC: semiannual percentage changes; 95%CI: 95% confidence interval.

Of all IGR, only eight (1.56%) had no cases of tuberculosis in people with HIV: Caracaraí, in Roraima; Tocantinópolis, in Tocantins; Simplício Mendes, São João do Piauí, and Canto do Buriti, in Piauí; Águas Formosas, in Minas Gerais; Cerro Largo, in Rio Grande do Sul; and Pires do Rio, in Goiás. The socioeconomic and programmatic indicators of all 510 regions were described in terms of arithmetic mean and SD and tested in regression models, as presented in Table 2.

**Table 2:** Descriptive measures and bivariate analyses of contextual indicators associated with treatment interruption and death from tuberculosis in people with HIV, Brazil, 2015–2021.

Parameter	Mean (SD)	Interruption	Death
		RR (95%CI)	RR (95%CI)
GINI	0.52 (0.04)	1.45 (1.28 to 1.63)	1.21 (1.12 to 1.31)
PER_OCUPEF	50.97 (10.24)	1.68 (1.54 to 1.84)	1.50 (1.41 to 1.60)
PER_DOMIC	81.16 (18.21)	1.23 (1.09 to 1.39)	1.17 (1.08 to 1.27)
PER_DENSI	26.68 (12.15)	1.21 (1.05 to 1.40)	1.10 (1.01 to 1.20)
ESP_VIDA	73.47 (2.22)	1.34 (1.19 to 1.52)	1.22 (1.13 to 1.32)
IDHM	0.68 (0.06)	1.50 (1.35 to 1.67)	1.35 (1.25 to 1.45)
RAZ_DEPEN	50.69 (8.42)	0.72 (0.65 to 0.80)	0.79 (0.74 to 0.85)
RENDA	561.82 (230.45)	1.59 (1.44 to 1.74)	1.41 (1.32 to 1.50)
PER_VULPOB	41.41 (19.69)	0.72 (0.64 to 0.82)	0.79 (0.73 to 0.86)
PER_POB	21.62 (15.25)	0.74 (0.65 to 0.83)	0.80 (0.74 to 0.86)
EXP_ESTU	9.46 (0.80)	1.18 (1.03 to 1.36)	1.13 (1.03 to 1.23)
PER_25EF	38.89 (10.08)	1.73 (1.59 to 1.89)	1.52 (1.43 to 1.62)
TX_ENVEL	7.73 (1.83)	0.80 (0.70 to 0.92)	0.85 (0.78 to 0.93)
PER_SAUSUPL	11.65 (10.25)	1.80 (1.62 to 1.99)	1.54 (1.44 to 1.65)
PER_ICSAP	23.45 (6.71)	0.59 (0.52 to 0.67)	0.68 (0.63 to 0.74)
PER_CADPBF	61.86 (15.18)	1.01 (0.87 to 1.17)	0.95 (0.87 to 1.04)
PER_IDRSAI	2.98 (2.77)	0.67 (0.60 to 0.75)	0.74 (0.69 to 0.81)
TX_AIDS	14.33 (31.04)	1.08 (0.93 to 1.26)	1.02 (0.94 to 1.11)
TX_MORT	6.36 (1.21)	1.06 (0.93 to 1.20)	1.03 (0.94 to 1.12)
TX_ANALF	15.58 (9.10)	0.68 (0.61 to 0.76)	0.75 (0.70 to 0.81)
TX_MORTDCNT	348.59 (91.07)	1.02 (0.89 to 1.16)	1.00 (0.92 to 1.09)
TX_MORTINF	12.85 (4.21)	0.93 (0.81 to 1.08)	0.95 (0.87 to 1.04)

SD: standard deviation; RR: rate ratio; 95%CI: 95% confidence interval; GINI: Gini index; PER_OCUPEF: percentage of employed individuals with completed primary education; PER_DOMIC: percentage of the population living in households with a bathroom and piped water; PER_DENSI: percentage of the population living in households with more than two persons per bedroom; ESP_VIDA: life expectancy at birth; IDHM: municipal human development index; RAZ_DEPEN: dependency ratio; RENDA: per capita income; PER_VULPOB: percentage vulnerable to poverty; PER_POB: percentage of the poor; EXP_ESTU: expected years of schooling at age 18; PER_25EF: percentage of individuals aged 25 years or older with completed primary education; TX_ENVEL: aging rate; PER_SAUSUPL: percentage covered by private health insurance; PER_ICSAP: percentage of hospitalizations for ambulatory care-sensitive conditions; PER_CADPBF: percentage of individuals enrolled in the Unified Registry who receive benefits from the Bolsa Família Program; PER_IDRSAI: percentage of hospitalizations for diseases related to inadequate sanitation; TX_AIDS: AIDS incidence rate; TX_MORT: crude mortality rate; TX_ANALF: illiteracy rate (≥ 18 years old); TX_MORTDCNT: mortality rate from noncommunicable diseases; TX_MORTINF: infant mortality rate.

In the final adjusted model, the following were associated with treatment interruption: Gini index (RR = 1.19; 95%CI: 1.08 to 1.32), employed individuals with primary education (RR = 1.38; 95%CI: 1.22 to 1.56), schooling expectancy (RR = 0.72; 95%CI: 0.61 to 0.86), and hospitalizations for ambulatory care-sensitive conditions (HACSC) (RR = 0.71; 95%CI: 0.63 to 0.79). For death, the associated factors were: households with more than two persons per bedroom (RR = 1.43; 95%CI: 1.33 to 1.55), supplementary health coverage (RR = 1.62; 95%CI: 1.50 to 1.75), and HACSC (RR = 0.80; 95%CI: 0.74 to 0.87).

Based on these variables, cluster analyses were conducted for each population stratum (< 100,000 inhabitants; 100,000–300,000 inhabitants; and > 300,000 inhabitants). Comparing group profiles within each stratum, stratum I had the highest mean tuberculosis mortality (27.47%) and HACSC (26.40%). Stratum II had the highest mean cure rate (61.46%), with high household density (26.90%). Stratum III had the highest treatment interruption rate (16.49%), as well as the highest schooling expectancy (9.59 years) (Table 3).

**Table 3:** Descriptive measures of tuberculosis outcomes in people with HIV and contextual indicators, according to clusters derived from the k-means method, Brazil, 2015–2021.

Parameter	Cluster						Total	
	Mean	SD	Mean	SD	Mean	SD	Mean	SD
< 100,000 inhabitants	Group I.I		Group I.II		Group I.III		Stratum I	
TX_CURA	44.66	22.02	70.58	30.55	15.00	23.35	54.46	32.39
TX_OBITO	37.53	23.35	9.01	12.43	77.00	28.65	27.47	29.16
TX_INTER	9.70	11.91	9.77	15.97	7.62	16.61	9.56	14.60
GINI	0.48	0.03	0.53	0.05	0.60	0.08	0.52	0.06
PER_OCUPEF	52.84	6.42	44.52	8.00	40.65	6.95	47.39	8.60
PER_DENSI	16.30	7.08	27.74	12.99	50.88	17.48	25.70	15.52
EXP_ESTU	9.98	0.70	9.21	0.78	8.02	0.91	9.38	0.97
PER_SAUSUPL	12.59	8.55	4.44	3.55	1.92	1.17	7.38	7.32
PER_ICSAP	22.79	6.09	28.70	7.84	29.01	8.74	26.40	7.87
100,000–300,000 inhabitants	Group II.I		Group II.II		Group II.III		Stratum II	
TX_CURA	71.13	14.22	49.28	15.96	61.91	13.02	61.46	16.41
TX_OBITO	21.25	12.09	29.19	14.25	25.82	12.83	25.29	13.34
TX_INTER	7.62	8.54	21.53	10.27	12.27	9.55	13.25	10.83
GINI	0.53	0.03	0.55	0.03	0.48	0.03	0.51	0.04
PER_OCUPEF	42.95	5.38	42.50	5.40	57.58	5.83	49.16	9.23
PER_DENSI	29.44	7.95	37.97	12.53	18.44	5.74	26.90	11.80
EXP_ESTU	9.13	0.44	8.82	0.70	9.98	0.60	9.42	0.77
PER_SAUSUPL	4.61	3.27	3.48	3.00	17.42	8.11	9.85	8.82
PER_ICSAP	22.66	5.58	29.58	7.39	22.15	4.70	24.24	6.59
> 300,000 inhabitants	Group III.I		Group III.II		Group III.III		Stratum III	
TX_CURA	61.95	11.38	61.39	10.65	59.20	10.53	60.73	10.90
TX_OBITO	23.23	8.44	22.36	6.05	22.78	7.88	22.78	7.54
TX_INTER	14.82	10.73	16.25	9.19	18.02	10.08	16.49	10.09
GINI	0.54	0.03	0.48	0.03	0.53	0.04	0.52	0.04
PER_OCUPEF	43.35	5.69	65.69	5.27	59.88	5.62	56.77	10.68
PER_DENSI	33.90	9.94	18.45	5.35	28.49	8.00	26.89	10.09
EXP_ESTU	8.89	0.51	10.25	0.47	9.58	0.44	9.59	0.72
PER_SAUSUPL	5.16	3.51	29.39	8.20	18.24	6.65	17.89	11.55
PER_ICSAP	23.51	4.30	18.41	3.45	18.57	2.97	20.01	4.24

SD: standard deviation; TX_CURA: cure rate; TX_OBITO: death rate; TX_INTER: treatment interruption rate; GINI: Gini index; PER_OCUPEF: percentage of employed individuals with completed primary education; PER_DENSI: percentage of the population living in households with more than two persons per bedroom; EXP_ESTU: expected years of schooling at age 18; PER_SAUSUPL: percentage covered by private health insurance; PER_ICSAP: percentage of hospitalizations for ambulatory care-sensitive conditions.

AC: Acre; AL: Alagoas; AP: Amapá; AM: Amazonas; BA: Bahia; CE: Ceará; ES: Espírito Santo; GO: Goiás; MA: Maranhão; MT: Mato Grosso; MS: Mato Grosso do Sul; MG: Minas Gerais; PA: Pará; PB: Paraíba; PR: Paraná; PE: Pernambuco; PI: Piauí; RJ: Rio de Janeiro; RN: Rio Grande do Norte; RS: Rio Grande do Sul; RO: Rondônia; RR: Roraima; SC: Santa Catarina; SP: São Paulo; SE: Sergipe; TO: Tocantins; DF: Federal District.

Group I.I had an average of 67,058 inhabitants and 261 municipalities; in stratum I, it showed the best indicators for schooling expectancy, supplementary health coverage, and workers with primary education. Scenario I.II had a mean population of 69,273 across 319 municipalities and showed the highest cure and treatment interruption rates. Cluster I.III had a mean population of 69,781 across 50 municipalities and recorded the highest death rate in stratum I, along with higher Gini index, household density, and HACSC (Table 3).

Group II.I included an average of 174,217 inhabitants across 801 municipalities; in stratum II, it had the highest mean cure rate. Cluster II.II comprised an average of 182,628 inhabitants across 645 municipalities and showed higher rates of death and interruption, along with higher Gini index, household density, and HACSC. Scenario II.III had an average of 180,991 inhabitants across 1,247 municipalities and recorded the highest percentages of workers with primary education and supplementary health coverage, as well as higher schooling expectancy (Table 3).

Cluster III.I had a mean population of 688,886 across 688 municipalities; in stratum III, it had the highest cure and death rates, as well as the highest Gini index, household density, and HACSC. Scenario III.II had an average population of 1,272,180 across 812 municipalities and recorded higher household density, schooling expectancy, and supplementary health coverage. Group III.III had an average of 1,102,696 inhabitants across 747 municipalities and showed the highest mean treatment interruption rate (Table 3).

The North region had the greatest representation of group I.I, along with other groups from stratum I. The Midwest region appeared to concentrate more groups from stratum I, with some incursions from stratum II. The South region, particularly Rio Grande do Sul and Paraná, had a higher predominance of stratum III groups, such as III.I and III.III. The Northeast region showed greater diversity between strata II and III, with some stratum I areas depending on the state. The Southeast region presented a combination of strata II and III, with lower representation of stratum I (Figure).

**Figure:**
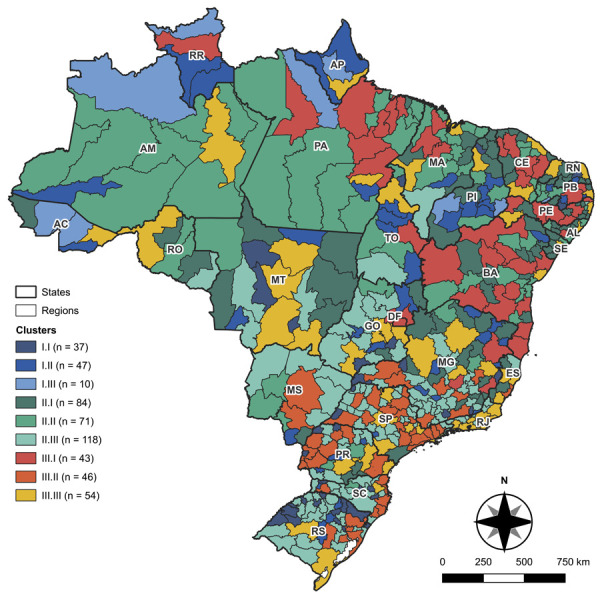
Spatial distribution of clusters of immediate geographic regions, according to clusters derived from the k-means method by population stratum of these regions, Brazil, 2015–2021.

## DISCUSSION

This study described trends in tuberculosis treatment outcomes among people with HIV in Brazil. Although cure rates remained predominantly stable over the study period, increasing temporal trends in treatment interruption and death were identified in some states. Moreover, patterns of inequality in the clustering of immediate regions, based on socioeconomic and programmatic indicators related to these outcomes, were revealed across Brazil.

The stability of cure rates, concomitant with increases in treatment interruption and death, suggests a likely reduction in the proportion of outcomes not considered in this research, which refer to operational records in SINAN, such as transfer and treatment regimen change. This decrease may reflect improvements in data classification and reporting, as well as changes in the epidemiological profile of the population. This aspect is crucial for understanding outcome dynamics and may be further detailed in future investigations.

States that experienced increases in treatment interruption and death may benefit from targeted care strategies. In Brazil, the use of antiretroviral therapy for all people with HIV is recommended as a priority strategy to improve treatment outcomes and, consequently, to reduce associated issues. In addition, the concomitant use of antiretroviral therapy during tuberculosis treatment has been shown to reduce mortality and improve clinical outcomes in people with HIV^17^.

Nationally, the study by Resende et al. demonstrated that HIV viral load reduction and the use of antiretroviral therapy were the main drivers of the decline in tuberculosis burden^18^. In this regard, the appropriate implementation of antiretroviral therapy may be crucial for the survival of people with tuberculosis coinfection, by restoring immune function and preventing the most severe forms of the disease^19^. Nevertheless, only 64.2% of all coinfection cases received antiretroviral therapy in Brazil in 2020^6^.

The findings linked the incidence of unfavorable tuberculosis treatment outcomes to socioeconomic inequalities; the Gini index increased treatment interruption by 19% (95%CI: 8% to 32%). In Latin America, a scoping review indicated that the main determinants of inequities in access to health services are income, education, transport, and housing^20^. This highlights the possible implications of social conditions for tuberculosis treatment in Brazilian regions.

Paradoxically, areas with high rates of HACSC, which reflect poor primary care performance, showed lower percentages of treatment interruption and death from tuberculosis among people with HIV. It is inferred that hospitalizations may function as a priority care point in contexts of weak primary care. In Brazil, average primary healthcare coverage is 80%, with disparities between geographic regions, signaling barriers to expanding access to basic care^21^.

Although tuberculosis treatment for people with HIV is recommended to be shared with specialized care services^17^, primary care plays a crucial role in establishing continuity and adherence to drug therapies^22^. Thus, HACSC, including tuberculosis, may be understood as a compensatory mechanism within the healthcare network—especially in HIV cases, for which decentralization from the secondary level remains incipient.

Regions with higher percentages of individuals employed with only primary education showed higher levels of treatment interruption, whereas those with higher expected years of schooling performed better. In a Chilean cohort, individuals with a university degree had a lower likelihood of death from coinfection^23^. This underscores that guaranteeing access to education in immediate regions may serve as a complementary approach to reducing disparities in access and adherence to tuberculosis treatment in Brazil.

The occurrence of negative outcomes was also higher in areas with greater household crowding (more than two people per bedroom). This indicator indirectly reflects the socioeconomic profile of the population, suggesting high household vulnerability^24^. Densely occupied households are associated with poverty, which is linked to malnutrition, comorbidities, and poor living conditions; these factors facilitate tuberculosis transmission and worsen the clinical course in people with HIV coinfection^25^.

A higher percentage of private health insurance coverage increased the probability of death from tuberculosis among people with HIV by 62% (95%CI: 50% to 75%). Fragmentation between public and private systems may result in gaps in the follow-up of people with coinfection. Furthermore, private coverage is generally concentrated in larger, better-developed regions^26^. In this sense, the inequalities generated in these territories may hinder access to treatment, leading to worse outcomes.

Given these findings, strategies based on strengthening care linkage are crucial to improve treatment follow-up among people with dual infection. Tools capable of reinforcing this connection with health services, such as directly observed therapy (DOT), should be considered. A study in a region of Malaysia found that people with HIV who did not receive DOT were more likely to discontinue treatment or die from tuberculosis (odds ratio – OR = 21.71; 95%CI: 5.36 to 87.94)^27^.

Since the socioeconomic context of regions was an important factor linked to treatment, actions focused on promoting access and continuity of care in socioeconomically vulnerable areas are indispensable. Specifically for tuberculosis treatment, receipt of financial incentives via government cash transfer programs has already been shown to be negatively associated with treatment discontinuation in a SINAN cohort (OR = 0.64; 95%CI: 0.50 to 0.81)^28^.

Nevertheless, in the Brazilian context, most tuberculosis cases are concentrated in large municipalities with poor performance in operational indicators, including insufficient HIV testing, poor DOT implementation, and low primary care coverage^29,30^. In this regard, structural policies must ensure healthcare within a broader context, involving measures to mitigate barriers and inequities in access and adherence to treatment, in light of the social and health inequalities evidenced in this study.

It should be noted that the use of secondary data is the major limitation of this study, given the possibility of underreporting. These data represent only those who accessed the health system; therefore, underrepresentation of people living in more vulnerable areas is possible. However, probabilistic linkage across four information systems (SINAN, SIM, SICLOM, and SISCEL) was employed to improve data quality, particularly with respect to correcting underreporting and qualifying outcomes.

Despite stable tuberculosis cure rates among people with HIV, increases in treatment interruption and death were observed. Regional disparities in the relationship between socioeconomic and programmatic indicators and tuberculosis outcomes suggest inequities in access to and adherence with treatment in different contexts across the country. Strengthening intersectoral policies focused on access and adherence to care is required in order to achieve the goal of eliminating these infections by 2030.

The contribution of this study lies in the description of trends in unfavorable tuberculosis treatment outcomes among people with HIV. The identification of clusters highlighted the influence of inequalities in access and linkage to care. Thus, this investigation may support the development of policies to strengthen the healthcare network and mitigate socioeconomic disparities. Further studies are needed to better understand the associations observed.
