# Supplementary material for: Temporal trends and regional clusters of tuberculosis-HIV coinfection outcomes in Brazil
Source: Rev Saude Publica. 2025 Nov 5;59:e39. doi: 10.11606/s1518-8787.2025059006736 (PMC12628741; doi:10.11606/s1518-8787.2025059006736)
Supplement: Translation

# Translation: Tendências temporais e agrupamentos regionais dos desfechos da coinfecção tuberculose-HIV no Brasil

**Authors:** Lucas Vinícius de Lima, Gabriel Pavinati, Yenny Lorena Valdivia-Rojas, José Nildo de Barros Silva, Ana Paula Sayuri Sato, Fredi Alexander Diaz-Quijano, Victor Santana Santos, Gabriela Tavares Magnabosco

**Affiliations:** I Brasil Universidade Estadual de Maringá. Centro de Ciências da Saúde. Programa de Pós-Graduação em Enfermagem. Maringá, PR, Brasil; II Brasil Prefeitura Municipal de Maringá. Secretaria Municipal de Saúde. Gerência de Planejamento. Maringá, PR, Brasil; III Chile Universidad de Antofagasta. Departamento de Enfermería. Unidad Académica de Enfermería. Antofagasta, AN, Chile; IV Brasil Universidade de São Paulo. Escola de Enfermagem de Ribeirão Preto. Programa de Pós-Graduação em Enfermagem em Saúde Pública. Ribeirão Preto, SP, Brasil; V Brasil Universidade de São Paulo. Faculdade de Saúde Pública. Departamento de Epidemiologia. São Paulo, SP, Brasil; VI Brasil Universidade Federal de Sergipe. Departamento de Medicina. Programa de Pós-Graduação em Ciências Aplicadas à Saúde. Lagarto, SE, Brasil

**Keywords:** HIV, Tuberculose, Estudos Ecológicos, Estudos de Séries Temporais, Análise por Conglomerados

## Abstract

**OBJETIVO:**

Investigar as tendências temporais e os agrupamentos regionais dos desfechos do tratamento da tuberculose em pessoas com HIV no Brasil, considerando sua relação com indicadores socioeconômicos e programáticos.

**MÉTODOS:**

Estudo ecológico com dados de pessoas vivendo com HIV que iniciaram e encerraram o tratamento para tuberculose entre 2015 e 2021 no Brasil. Foram descritas as tendências semestrais das taxas de cura, interrupção do tratamento e óbito nos estados brasileiros, usando-se modelos de regressão *joinpoint*. Análises de *cluster*, estratificadas em três portes populacionais, foram realizadas pelo método *k-means* visando identificar agrupamentos nas 510 regiões geográficas imediatas. Empregaram-se indicadores socioeconômicos e programáticos relacionados à interrupção do tratamento e ao óbito, mediante associação em modelos multivariados de regressão binomial negativa.

**RESULTADOS:**

Analisaram-se 54.362 tratamentos de tuberculose em pessoas com HIV, com taxas de cura em 55,51%, interrupção em 23,33% e óbito em 21,16%, para o período. Observou-se estabilidade na cura a nível nacional, enquanto a interrupção do tratamento aumentou semestralmente em 2,54% (variando entre 1,59% e 3,70%) e o óbito em 9,31% (oscilando entre 7,41% e 17,24%). Estados como Ceará e Amapá apresentaram as piores tendências para as taxas de interrupção do tratamento e óbito. Regiões com maiores desigualdades na distribuição de renda, percentuais de trabalhadores com ensino fundamental, percentuais de densidade domiciliar e coberturas da saúde suplementar tiveram elevadas taxas de interrupção do seguimento e óbito. Em contrapartida, aquelas com maiores expectativas de anos de estudo e internações por condições sensíveis à atenção primária apresentaram menores probabilidades desses desfechos.

**CONCLUSÃO:**

Nacionalmente, apesar da estabilidade na cura, viu-se aumento nas taxas de interrupção do tratamento e óbito por tuberculose em pessoas com HIV. As disparidades regionais na relação de indicadores socioeconômicos e programáticos com os desfechos sugerem iniquidades no acesso e na adesão ao tratamento da tuberculose nos diferentes territórios do país.

## INTRODUÇÃO

A coinfecção por tuberculose em pessoas vivendo com vírus da imunodeficiência humana (HIV) representa um dos maiores desafios de saúde pública. A tuberculose, historicamente associada a condições de vulnerabilidade socioeconômica, ganha uma complexidade adicional com o HIV pela imunossupressão envolvida^1^. Segundo a Organização Mundial da Saúde (OMS)^2^, estima-se que 10,8 milhões de pessoas adoeceram por tuberculose no mundo em 2023, das quais aproximadamente 6,1% foram indivíduos com HIV^2^.

No Brasil, em 2023, foram registrados 80 mil casos novos de tuberculose; destes, calcula-se que 9,3% se tratavam de pessoas com coinfecção pelo HIV^4^. Na série de 2010 a 2021, houve uma redução anual de -4,3% (variação entre -5,1% e -3,7%) na incidência da dupla infecção^5^. O país persiste com uma significativa carga desses agravos e tem implementado diversas políticas para controlar a tuberculose em pessoas com HIV; todavia, entraves relacionados à conclusão do tratamento parecem dificultar a resposta nacional^6,7^.

O enfrentamento da coinfecção tuberculose-HIV é uma prioridade para a agenda global, sendo diretamente abordado nos Objetivos de Desenvolvimento Sustentável da Organização das Nações Unidas, que visam à eliminação da tuberculose e à redução do impacto do HIV até 2030^8^. Para atingir essa meta, os países devem fortalecer seus sistemas de saúde por meio de financiamento adequado^8^, garantindo o acesso universal a diagnósticos precoces e tratamentos oportunos, especialmente para populações vulneráveis.

No contexto brasileiro, as taxas de cura ficaram em torno de 50,6% em 2020, com melhorias entre pessoas em uso de terapia antirretroviral e com contagem de linfócitos CD4+ > 350 cél./mm^3^; já a interrupção do tratamento e o óbito totalizaram 16,9% e 21,8% dos encerramentos, respectivamente^6^. Estudos indicam que interações e reações medicamentosas, restrições financeiras, fragilidades dos serviços de saúde e vulnerabilidades sociais atreladas às infecções podem dificultar a conclusão da terapia antituberculose nas pessoas com HIV^7,9^.

A agenda para 2030 exige uma abordagem que considere esses entraves de forma intersetorial, visando otimizar as respostas da terapêutica da coinfecção no país. Para tanto, deve-se identificar padrões na ocorrência dos desfechos do tratamento, revelando áreas que requerem ações estratégicas. Diante disso, este estudo investigou as tendências temporais e os agrupamentos regionais dos desfechos do tratamento da tuberculose em pessoas com HIV no Brasil, considerando sua relação com indicadores socioeconômicos e programáticos.

## MÉTODOS

É um estudo ecológico com dados de vigilância acerca do tratamento da tuberculose em pessoas com HIV no Brasil provenientes de sistemas de informação em saúde. Embora tenham sido utilizados dados agregados, concedidos pelo Ministério da Saúde mediante solicitação na Plataforma Fala.BR (protocolo n.º 25072.039887/2022-27), obteve-se aprovação pelo Comitê de Ética em Pesquisa (parecer n.º 6.914.233/2024) por integrar uma pesquisa acadêmica com múltiplos métodos, consoante a Resolução n.º 674/2022, do Conselho Nacional de Saúde.

A abrangência deste estudo foi o Brasil, que possui área aproximada de 8,5 mil km^2^ e conta com cerca de 203 milhões de pessoas^12^. O Brasil, maior país na região da América Latina, dispõe de 5.570 municípios e distritos, que se organizam, administrativamente, em 27 unidades federativas. Em um nível menor de agregação, eles formam 510 regiões geográficas imediatas (RGI), considerando a conexão de cidades por meio de relações de dependência e deslocamento da população para suprir as necessidades imediatas, como saúde, educação e emprego.

Os dados foram provenientes de *linkage* probabilístico, conforme a metodologia apresentada pelo Ministério da Saúde^6^, entre o Sistema de Informação de Agravos de Notificação (Sinan), o Sistema de Informação sobre Mortalidade (SIM), o Sistema de Controle Logístico de Medicamentos (Siclom) e o Sistema de Controle de Exames Laboratoriais da Rede Nacional de Contagem de Linfócitos CD4+/CD8+ e Carga Viral do HIV (Siscel). Foram realizadas três relações: (i) entre as bases do HIV; (ii) entre as bases da tuberculose; e (iii) entre os produtos dessas etapas^6^.

A população consistiu nas pessoas com HIV que iniciaram o tratamento da tuberculose de janeiro de 2015 a junho de 2021, e o concluíram até dezembro de 2021. Incluíram-se aquelas com as variáveis “HIV” ou “aids” codificadas como “sim” no Sinan da tuberculose, ou aquelas sem esse preenchimento, mas que tinham registro no Sinan-HIV, SIM-HIV, Siclom ou Siscel^6^. Os casos novos, de recidiva ou de retratamento de tuberculose com a “situação de encerramento” não preenchida ou preenchida com desfechos operacionais (9%) foram excluídos.

A variável principal foi extraída da “situação de encerramento” no Sinan, sendo: “cura”, que consiste na conclusão/alta do tratamento da tuberculose; “abandono primário” e “abandono” (i.e., “interrupção do tratamento”), que representam, respectivamente, a interrupção do tratamento antes e após 30 dias de seu início, com ausência por 30 dias consecutivos; e “óbito por tuberculose” e “óbito por outras causas” (i.e., “óbito”), que contemplam, respectivamente, as mortes com tuberculose mencionada como causa básica e associada no SIM.

As variáveis secundárias foram extraídas do Atlas do Desenvolvimento Humano no Brasil (Atlas Brasil)^13^, uma plataforma on-line que disponibiliza dados sobre múltiplos indicadores – mais de 300 – que refletem as condições de vida no país nas dimensões social, econômica, política e ambiental. Foi selecionado, a partir de estudos prévios e hipóteses, um rol de indicadores socioeconômicos e programáticos dos municípios brasileiros, também referidos como contextuais neste estudo, devidamente apresentados noQuadro.

**Quadro:** Descrição dos indicadores socioeconômicos e programáticos (contextuais), com mensuração referente aos municípios do país, selecionados para compor este estudo, Brasil, 2024.

Código	Descrição	Fonte
GINI	Índice de Gini: mede o grau de desigualdade existente na distribuição de indivíduos segundo a renda domiciliar per capita; seu valor varia de 0,00 (quando não há desigualdade, isto é, a renda domiciliar per capita de todos os indivíduos têm o mesmo valor), a 1,00 (quando a desigualdade é máxima, isto é, apenas um indivíduo detém toda a renda).	IBGE (2010)
PER_OCUPEF	Percentual dos ocupados com ensino fundamental completo: razão entre o número de pessoas de 18 anos ou mais de idade ocupadas que já concluíram o ensino fundamental e o número total de pessoas ocupadas nessa faixa etária, multiplicada por 100.	IBGE (2010)
PER_DOMIC	Percentual da população que vive em domicílios com banheiro e água encanada: razão entre a população que vive em domicílios particulares permanentes com água encanada em pelo menos um de seus cômodos e com banheiro exclusivo e a população total residente em domicílios particulares permanentes, multiplicada por 100.	IBGE (2010)
PER_DENSI	Percentual da população que vive em domicílios com densidade superior a duas pessoas por dormitório: razão entre a população que vive em domicílios particulares permanentes com densidade superior a duas pessoas por dormitório e a população total residente em domicílios particulares permanentes, multiplicada por 100.	IBGE (2010)
ESP_VIDA	Esperança de vida ao nascer: número médio de anos que as pessoas deverão viver a partir do nascimento, se permanecerem constantes ao longo da vida o nível e o padrão de mortalidade por idades prevalecentes no ano do censo.	IBGE (2010)
IDHM	Índice de desenvolvimento humano municipal: média geométrica dos índices atribuíveis às dimensões de renda, educação e longevidade, com pesos iguais; varia de 0,00 a 1,00, do baixo ao muito alto desenvolvimento, respectivamente.	IBGE (2010)
RAZ_DEPEN	Razão de dependência: razão entre o número de pessoas com 14 anos ou menos e de 65 anos ou mais de idade (população dependente) e o número de pessoas com idade de 15 a 64 anos (população potencialmente ativa), multiplicada por 100.	IBGE (2010)
RENDA	Renda per capita: razão entre o somatório da renda de todos os indivíduos residentes em domicílios particulares permanentes e o número total desses indivíduos.	IBGE (2010)
PER_VULPOB	Percentual de vulneráveis à pobreza: proporção dos indivíduos com renda domiciliar per capita igual ou inferior a 255,00 reais mensais, equivalente a meio salário mínimo na data.	IBGE (2010)
PER_POB	Percentual de pobres: proporção dos indivíduos com renda domiciliar per capita igual ou inferior a 140,00 reais mensais.	IBGE (2010)
EXP_ESTU	Expectativa de anos de estudo aos 18 anos de idade: número médio de anos de estudo que uma geração de crianças que ingressa na escola deverá completar ao atingir 18 anos de idade, se os padrões atuais se mantiverem ao longo de sua vida escolar.	IBGE (2010)
PER_25EF	Percentual de pessoas de 25 anos ou mais de idade com ensino fundamental completo: razão entre a população de 25 anos ou mais de idade que concluiu o ensino fundamental, em quaisquer de suas modalidades, e o total de pessoas nesta faixa etária, multiplicada por 100.	IBGE (2010)
TX_ENVEL	Taxa de envelhecimento: razão entre a população de 65 anos ou mais de idade e a população total, multiplicada por 100.	IBGE (2010)
PER_SAUSUPL	Percentual de pessoas cobertas por planos de saúde suplementar: participação percentual da população coberta por planos de saúde suplementar no total da população.	SIB (2017)
PER_ICSAP	Percentual de internações por condições sensíveis à atenção primária: participação percentual de internações hospitalares por condições sensíveis à atenção primária no total de internações hospitalares.	SIM (2017)
PER_CADPBF	Percentual de pessoas inscritas no Cadastro Único que recebem pelo Programa Bolsa Família: participação percentual de pessoas inscritas no Cadastro Único cuja família recebe Bolsa Família, no total de pessoas inscritas no Cadastro Único.	CadÚnico (2017)
PER_IDRSAI	Percentual de internações por doenças relacionadas ao saneamento ambiental inadequado: participação percentual de internações hospitalares por doenças relacionadas ao saneamento ambiental inadequado no total de internações hospitalares.	SIH/SUS (2017)
TX_AIDS	Taxa de incidência de aids: razão entre o número de casos novos de aids (códigos B20 a B24 da CID-10) registrados no ano considerado e a população total, multiplicada por 100.000	Sinan (2017)
TX_MORT	Taxa bruta de mortalidade: número total de óbitos no total da população, multiplicado por 100.000	SIM (2017)
TX_ANALF	Taxa de analfabetismo (18 anos ou mais de idade): razão entre a população de 18 anos ou mais de idade que não sabe ler nem escrever um bilhete simples e o total de pessoas nesta faixa etária, multiplicada por 100.	IBGE (2010)
TX_MORTDCNT	Taxa de mortalidade por doenças não transmissíveis: número de óbitos por doenças não transmissíveis no total da população, multiplicado por 100.000.	SIM (2017)
TX_MORTINF	Taxa de mortalidade infantil: razão entre o número de crianças que não deverão sobreviver ao primeiro ano de vida e o número de crianças nascidas vivas, multiplicada por 1.000.	IBGE (2010)

IBGE: Instituto Brasileiro de Geografia e Estatística; SIB: Sistema de Informações de Beneficiários; SIM: Sistema de Informação sobre Mortalidade; CadÚnico: Cadastro Único; SIH/SUS: Sistema de Informações Hospitalares do Sistema Único de Saúde; CID-10: Classificação Internacional de Doenças – 10ª edição; Sinan: Sistema de Informação de Agravos de Notificação.

Inicialmente, calcularam-se as taxas de cura, interrupção do tratamento e óbito, para cada estado, como a proporção entre o número de pessoas com o desfecho de interesse e o número total de pessoas notificadas naquele local; o resultado desta divisão foi multiplicado por 100. Devido ao recorte temporal disponível na base de dados concedida, optou-se por utilizar as taxas semestrais a partir da data de término do tratamento, ampliando o número de pontos a serem analisados; portanto, obtiveram-se 13 pontos (semestres) passíveis de estimativa de tendência.

A avaliação da tendência foi realizada por modelos de regressão *joinpoint*, apropriados para a análise de séries temporais por considerar a autocorrelação dos dados^14^. As taxas de cada desfecho, transformadas por função logarítmica natural devido à melhor interpretação e comparação, foram consideradas como a variável dependente, e os semestres-calendário do período como a variável independente. Os modelos log-lineares foram ajustados pelos erros-padrão das taxas e pela correção da autocorrelação de primeira ordem, se verificada.

Para os modelos finais de cada série, calcularam-se, por meio do método quantil-empírico, as variações percentuais semestrais (VPS), referentes à mudança relativa nos valores semestrais das taxas de cada desfecho ao longo do período analisado, e seus IC95% (intervalos de confiança de 95%). Foram considerados significativos os valores das variações semestrais cujos IC95% não incluíssem o valor nulo (0,00), indicando a tendência crescente ou decrescente das taxas. Para essas análises, utilizou-se o Joinpoint Regression Program® (versão 5.0.2).

As variáveis descritas no Quadroforam representadas pela média aritmética e pelo desvio-padrão (DP). Para identificar os fatores associados à interrupção do tratamento e ao óbito por tuberculose em pessoas com HIV, empregaram-se modelos de regressão com distribuição binomial negativa perante evidência de superdispersão dos dados^15^. Nesses modelos, a contagem do evento foi tratada como a variável dependente, os indicadores contextuais como as variáveis independentes e o total de casos na RGI correspondente como o *offset*.

Para os modelos de regressão, as variáveis independentes foram agrupadas nas 510 RGI por meio de média ponderada, tendo-se a população municipal do censo de 2022 como fator de ponderação. Essas variáveis foram padronizadas a partir da subtração pela média aritmética, seguida pela divisão desse resultado pelo DP, para facilitar a comparação entre magnitudes de associação. As medidas de associação dos modelos foram as razões de taxas (RT), consideradas significativas quando seus IC95% foram diferentes de 1,00 (valor nulo).

Avaliou-se cada variável independente em análises bivariadas, para cada um dos desfechos negativos. Para obter modelos múltiplos parcimoniosos, evitou-se incluir variáveis correlacionadas entre si (com sinais de multicolinearidade). Sempre que uma correlação entre variáveis foi identificada pelo coeficiente de Spearman, para o modelo múltiplo, selecionou-se a variável com a maior associação com o desfecho na avaliação bivariada. Empregou-se um método de seleção *backward stepwise*, preservando-se variáveis com valor de p < 0,05 nos modelos finais.

Com as variáveis do modelo final, realizou-se a análise de agrupamento não hierárquico pelo método *k-means*, que possibilita a divisão do conjunto de dados em *k* grupos, minimizando a soma das distâncias quadradas entre os pontos e seus respectivos centroides^16^. Neste estudo, o valor de *k* foi definido pelo método *elbow*. Como a variação do número de habitantes (hab.) entre as RGI é elevada, optou-se por proceder ao agrupamento mediante o estrato populacional das regiões: < 100 mil hab. (estrato I), 100–300 mil hab. (estrato II) e > 300 mil hab. (estrato III).

Para cada estrato populacional, a premissa é que os dados dentro de cada grupo gerado pela análise sejam mais semelhantes entre si do que aos dados de outros grupos no respectivo estrato, com base na distância entre os pontos e os centroides. Além disso, um mapa coroplético foi construído com auxílio do *software* QGIS® (versão 2.36.3), ilustrando o perfil dos grupos gerados pela análise de agrupamento em cada um dos estratos populacionais. As demais análises foram realizadas com apoio do *software* SPSS Statistics® (versão 25.0).

## RESULTADOS

Foram analisados 54.362 casos de tratamento da tuberculose em pessoas com HIV no Brasil. A taxa de cura foi 55,51%, ao passo que a de interrupção do tratamento foi 23,33%, e a de óbito, 21,16%, para todo o período. Na análise de tendência, observou-se série estacionária para as taxas de cura do tratamento no país, com variação semestral igual a -1,02% (IC95% -4,33% a 2,41%); a interrupção do tratamento e o óbito apresentaram aumento semestral de 2,54% (IC95% 1,59% a 3,70%) e 9,31% (IC95% 7,41% a 17,24%), respectivamente.

Notou-se redução semestral da cura de -3,24% (IC95% -5,83% a -0,82%) no Ceará e de -2,39% (IC95% -4,13% a -1,35%) no Distrito Federal. Referente à interrupção do tratamento, nove unidades tiveram aumento, com maior intensidade no Amapá (VPS = 21,35%; IC95% 9,99% a 33,72%) e no Ceará (VPS = 5,09%; IC95% 2,18% a 8,94%). Quanto ao óbito, apenas o Espírito Santo apresentou declínio, de -14,68% (IC95% -21,44% a -6,94%); cinco estados registraram aumento, com destaque para o Ceará (VPS = 13,63%; IC95% 10,22% a 26,34%) (Tabela 1).

**Tabela 1:** Tendências temporais semestrais das taxas de cura, interrupção do tratamento e óbito por tuberculose em pessoas com HIV, segundo as unidades federativas, Brasil, 2015–2021.

Localidade	Cura	Interrupção	Óbito
	VPS (IC95%)	VPS (IC95%)	VPS (IC95%)
Norte			
Rondônia	-1,56 (-4,06 a 0,50)	4,84 (1,67 a 8,26)	-3,64 (-12,13 a 4,57)
Acre	0,04 (-0,27 a 0,46)	-0,00 (-0,29 a 0,12)	-5,46 (-15,05 a 5,36)
Amazonas	0,30 (-3,09 a 3,84)	2,97 (-0,01 a 5,46)	9,00 (-6,80 a 26,50)
Roraima	0,41 (-2,51 a 2,77)	-2,95 (-16,52 a 16,40)	3,52 (-1,13 a 9,02)
Pará	-1,03 (-3,22 a 1,48)	4,32 (2,09 a 6,84)	9,09 (2,82 a 19,22)
Amapá	-1,94 (-5,01 a 0,94)	21,35 (9,99 a 33,72)	1,18 (-5,81 a 10,09)
Tocantins	2,74 (-3,76 a 12,39)	3,51 (-9,12 a 17,56)	4,35 (-13,10 a 15,64)
Nordeste			
Maranhão	0,13 (-2,03 a 2,70)	0,35 (-2,44 a 3,29)	3,17 (-1,49 a 8,65)
Piauí	-2,04 (-5,12 a 0,58)	-1,87 (-7,83 a 3,53)	11,11 (7,09 a 16,83)
Ceará	-3,24 (-5,83 a -0,82)	5,09 (2,18 a 8,94)	13,63 (10,22 a 26,34)
Rio Grande do Norte	-1,03 (-5,52 a 4,05)	-0,97 (-11,48 a 11,20)	3,55 (-3,45 a 11,40)
Paraíba	-2,44 (-7,79 a 2,81)	3,88 (-4,22 a 14,21)	2,44 (-7,19 a 15,12)
Pernambuco	-0,58 (-3,29 a 2,31)	3,58 (1,12 a 5,97)	4,70 (-1,73 a 12,85)
Alagoas	-2,23 (-7,68 a 2,33)	3,68 (-0,79 a 8,92)	5,76 (-3,43 a 16,36)
Sergipe	0,22 (-1,89 a 2,32)	1,93 (-2,09 a 7,01)	-1,15 (-10,30 a 9,12)
Bahia	-0,25 (-2,00 a 1,52)	-0,43 (-3,46 a 2,36)	2,99 (-1,96 a 7,87)
Sudeste			
Minas Gerais	1,38 (-1,15 a 3,89)	-1,67 (-4,35 a 0,61)	1,47 (-4,52 a 6,74)
Espírito Santo	-10,20 (-26,26 a 7,44)	-12,43 (-21,52 a 0,50)	-14,68 (-21,44 a -6,94)
Rio de Janeiro	-0,35 (-2,31 a 1,68)	2,41 (0,81 a 4,28)	2,81 (-1,16 a 6,83)
São Paulo	-0,65 (-1,89 a 0,51)	2,72 (1,72 a 3,73)	0,65 (-3,55 a 4,86)
Sul			
Paraná	-0,01 (-3,49 a 3,37)	3,20 (-3,39 a 9,93)	5,05 (-3,84 a 12,78)
Santa Catarina	-0,24 (-2,70 a 2,10)	-0,33 (-6,87 a 6,07)	9,22 (0,67 a 19,91)
Rio Grande do Sul	-0,26 (-3,74 a 3,33)	2,23 (0,07 a 4,44)	3,96 (-0,74 a 9,08)
Centro-Oeste			
Mato Grosso	2,28 (-1,42 a 6,38)	-1,57 (-5,16 a 1,51)	4,56 (-7,30 a 16,07)
Mato Grosso do Sul	-0,56 (-5,30 a 5,48)	1,17 (-1,97 a 4,74)	5,22 (0,07 a 13,55)
Goiás	1,34 (-3,77 a 6,33)	0,17 (-6,24 a 5,83)	0,84 (-4,66 a 5,50)
Distrito Federal	-2,39 (-4,13 a -1,35)	5,70 (2,02 a 9,56)	4,37 (-3,23 a 12,33)

VPS: variação percentual semestral; IC95% intervalo de confiança de 95%.

Da totalidade de RGI, apenas oito (1,56%) não tiveram casos de tuberculose em pessoas com HIV, sendo elas: Caracaraí, em Roraima; Tocantinópolis, no Tocantins; Simplício Mendes, São João do Piauí e Canto do Buriti, no Piauí; Águas Formosas, em Minas Gerais; Cerro Largo, no Rio Grande do Sul; e Pires do Rio, em Goiás. Os indicadores socioeconômicos e programáticos de todas as 510 regiões foram descritos em função da média aritmética e do DP, bem como testados em modelos de regressão, conforme a apresentação na Tabela 2.

**Tabela 2:** Medidas descritivas e análises bivariadas dos indicadores contextuais associados à interrupção do tratamento e ao óbito por tuberculose em pessoas com HIV, Brasil, 2015–2021.

Variável	Média (DP)	Interrupção	Óbito
		RT (IC95%)	RT (IC95%)
GINI	0,52 (0,04)	1,45 (1,28 a 1,63)	1,21 (1,12 a 1,31)
PER_OCUPEF	50,97 (10,24)	1,68 (1,54 a 1,84)	1,50 (1,41 a 1,60)
PER_DOMIC	81,16 (18,21)	1,23 (1,09 a 1,39)	1,17 (1,08 a 1,27)
PER_DENSI	26,68 (12,15)	1,21 (1,05 a 1,40)	1,10 (1,01 a 1,20)
ESP_VIDA	73,47 (2,22)	1,34 (1,19 a 1,52)	1,22 (1,13 a 1,32)
IDHM	0,68 (0,06)	1,50 (1,35 a 1,67)	1,35 (1,25 a 1,45)
RAZ_DEPEN	50,69 (8,42)	0,72 (0,65 a 0,80)	0,79 (0,74 a 0,85)
RENDA	561,82 (230,45)	1,59 (1,44 a 1,74)	1,41 (1,32 a 1,50)
PER_VULPOB	41,41 (19,69)	0,72 (0,64 a 0,82)	0,79 (0,73 a 0,86)
PER_POB	21,62 (15,25)	0,74 (0,65 a 0,83)	0,80 (0,74 a 0,86)
EXP_ESTU	9,46 (0,80)	1,18 (1,03 a 1,36)	1,13 (1,03 a 1,23)
PER_25EF	38,89 (10,08)	1,73 (1,59 a 1,89)	1,52 (1,43 a 1,62)
TX_ENVEL	7,73 (1,83)	0,80 (0,70 a 0,92)	0,85 (0,78 a 0,93)
PER_SAUSUPL	11,65 (10,25)	1,80 (1,62 a 1,99)	1,54 (1,44 a 1,65)
PER_ICSAP	23,45 (6,71)	0,59 (0,52 a 0,67)	0,68 (0,63 a 0,74)
PER_CADPBF	61,86 (15,18)	1,01 (0,87 a 1,17)	0,95 (0,87 a 1,04)
PER_IDRSAI	2,98 (2,77)	0,67 (0,60 a 0,75)	0,74 (0,69 a 0,81)
TX_AIDS	14,33 (31,04)	1,08 (0,93 a 1,26)	1,02 (0,94 a 1,11)
TX_MORT	6,36 (1,21)	1,06 (0,93 a 1,20)	1,03 (0,94 a 1,12)
TX_ANALF	15,58 (9,10)	0,68 (0,61 a 0,76)	0,75 (0,70 a 0,81)
TX_MORTDCNT	348,59 (91,07)	1,02 (0,89 a 1,16)	1,00 (0,92 a 1,09)
TX_MORTINF	12,85 (4,21)	0,93 (0,81 a 1,08)	0,95 (0,87 a 1,04)

DP: desvio-padrão; RT: razão de taxas; IC95% intervalo de confiança de 95%; GINI: índice de Gini; PER_OCUPEF: percentual dos ocupados com ensino fundamental completo; PER_DOMIC: percentual da população que vive em domicílios com banheiro e água encanada; PER_DENSI: percentual da população que vive em domicílios com densidade superior a duas pessoas por dormitório; ESP_VIDA: esperança de vida ao nascer; IDHM: índice de desenvolvimento humano municipal; RAZ_DEPEN: razão de dependência; RENDA: renda per capita; PER_VULPOB: percentual de vulneráveis à pobreza; PER_POB percentual de pobres; EXP_ESTU: expectativa de anos de estudo aos 18 anos de idade; PER_25EF: percentual de pessoas de 25 anos ou mais de idade com ensino fundamental completo; TX_ENVEL: taxa de envelhecimento; PER_SAUSUPL: percentual de pessoas cobertas por planos de saúde suplementar; PER_ICSAP: percentual de internações por condições sensíveis à atenção primária; PER_CADPBF: percentual de pessoas inscritas no Cadastro Único que recebem pelo Programa Bolsa Família; PER_IDRSAI: percentual de internações por doenças relacionadas ao saneamento ambiental inadequado; TX_AIDS: taxa de incidência de aids; TX_MORT: taxa bruta de mortalidade; TX_ANALF: taxa de analfabetismo (18 anos ou mais de idade); TX_MORTDCNT: taxa de mortalidade por doenças não transmissíveis; TX_MORTINF: taxa de mortalidade infantil.

No modelo ajustado final, associaram-se à interrupção do tratamento: índice de Gini (RT = 1,19; IC95% 1,08 a 1,32), ocupados com ensino fundamental (RT = 1,38; IC95% 1,22 a 1,56), expectativa de estudo (RT = 0,72; IC95% 0,61 a 0,86) e internações por condições sensíveis à atenção primária – ICSAP (RT = 0,71; IC95% 0,63 a 0,79). Em relação ao óbito, associaram-se: domicílios com densidade superior a duas pessoas (RT = 1,43; IC95% 1,33 a 1,55), cobertura de saúde suplementar (RT = 1,62; IC95% 1,50 a 1,75) e ICSAP (RT = 0,80; IC95% 0,74 a 0,87).

A partir dessas variáveis, foram realizadas as análises de agrupamento para cada estrato populacional (< 100 mil hab.; 100–300 mil hab.; e > 300 mil hab.). Ao comparar o perfil dos grupos em cada estrato, notou-se que o estrato I foi composto pela maior média de óbito por tuberculose (27,47%) e de ICSAP (26,40%). O estrato II teve a maior média de cura do tratamento (61,46%), com alta densidade domiciliar (26,90%). E o estrato III teve a maior taxa de interrupção do tratamento (16,49%), bem como alta expectativa de estudo (9,59 anos) (Tabela 3).

**Tabela 3:** Medidas descritivas dos desfechos da tuberculose em pessoas com HIV e dos indicadores contextuais, segundo os *clusters* provenientes do método *k*-*means*, Brasil, 2015–2021.

Variável	Agrupamento						Total	
	Média	DP	Média	DP	Média	DP	Média	DP
< 100 mil habitantes	Grupo I.I		Grupo I.II		Grupo I.III		Estrato I	
TX_CURA	44,66	22,02	70,58	30,55	15,00	23,35	54,46	32,39
TX_OBITO	37,53	23,35	9,01	12,43	77,00	28,65	27,47	29,16
TX_INTER	9,70	11,91	9,77	15,97	7,62	16,61	9,56	14,60
GINI	0,48	0,03	0,53	0,05	0,60	0,08	0,52	0,06
PER_OCUPEF	52,84	6,42	44,52	8,00	40,65	6,95	47,39	8,60
PER_DENSI	16,30	7,08	27,74	12,99	50,88	17,48	25,70	15,52
EXP_ESTU	9,98	0,70	9,21	0,78	8,02	0,91	9,38	0,97
PER_SAUSUPL	12,59	8,55	4,44	3,55	1,92	1,17	7,38	7,32
PER_ICSAP	22,79	6,09	28,70	7,84	29,01	8,74	26,40	7,87
100–300 mil habitantes	Grupo II.I		Grupo II.II		Grupo II.III		Estrato II	
TX_CURA	71,13	14,22	49,28	15,96	61,91	13,02	61,46	16,41
TX_OBITO	21,25	12,09	29,19	14,25	25,82	12,83	25,29	13,34
TX_INTER	7,62	8,54	21,53	10,27	12,27	9,55	13,25	10,83
GINI	0,53	0,03	0,55	0,03	0,48	0,03	0,51	0,04
PER_OCUPEF	42,95	5,38	42,50	5,40	57,58	5,83	49,16	9,23
PER_DENSI	29,44	7,95	37,97	12,53	18,44	5,74	26,90	11,80
EXP_ESTU	9,13	0,44	8,82	0,70	9,98	0,60	9,42	0,77
PER_SAUSUPL	4,61	3,27	3,48	3,00	17,42	8,11	9,85	8,82
PER_ICSAP	22,66	5,58	29,58	7,39	22,15	4,70	24,24	6,59
> 300 mil habitantes	Grupo III.I		Grupo III.II		Grupo III.III		Estrato III	
TX_CURA	61,95	11,38	61,39	10,65	59,20	10,53	60,73	10,90
TX_OBITO	23,23	8,44	22,36	6,05	22,78	7,88	22,78	7,54
TX_INTER	14,82	10,73	16,25	9,19	18,02	10,08	16,49	10,09
GINI	0,54	0,03	0,48	0,03	0,53	0,04	0,52	0,04
PER_OCUPEF	43,35	5,69	65,69	5,27	59,88	5,62	56,77	10,68
PER_DENSI	33,90	9,94	18,45	5,35	28,49	8,00	26,89	10,09
EXP_ESTU	8,89	0,51	10,25	0,47	9,58	0,44	9,59	0,72
PER_SAUSUPL	5,16	3,51	29,39	8,20	18,24	6,65	17,89	11,55
PER_ICSAP	23,51	4,30	18,41	3,45	18,57	2,97	20,01	4,24

DP: desvio-padrão; TX_CURA: taxa de cura; TX_OBITO: taxa de óbito; TX_INTER: taxa de interrupção do tratamento; GINI: índice de Gini; PER_OCUPEF: percentual dos ocupados com ensino fundamental completo; PER_DENSI: percentual da população que vive em domicílios com densidade superior a duas pessoas por dormitório; EXP_ESTU: expectativa de anos de estudo aos 18 anos de idade; PER_SAUSUPL: percentual de pessoas cobertas por planos de saúde suplementar; PER_ICSAP: percentual de internações por condições sensíveis à atenção primária.

AC: Acre; AL: Alagoas; AP: Amapá; AM: Amazonas; BA: Bahia; CE: Ceará; ES: Espírito Santo; GO: Goiás; MA: Maranhão; MT: Mato Grosso; MS: Mato Grosso do Sul; MG: Minas Gerais; PA: Pará; PB: Paraíba; PR: Paraná; PE: Pernambuco; PI: Piauí; RJ: Rio de Janeiro; RN: Rio Grande do Norte; RS: Rio Grande do Sul; RO: Rondônia; RR: Roraima; SC: Santa Catarina; SP: São Paulo; SE: Sergipe; TO: Tocantins; DF: Distrito Federal.

O grupo I.I foi composto por uma média de 67.058 hab. e 261 municípios; no estrato I, ele registrou os melhores indicadores de expectativa de estudo, cobertura de saúde suplementar e trabalhadores com ensino fundamental. O cenário I.II teve uma população média de 69.273 hab., com 319 municípios; ele registrou as maiores taxas de cura e interrupção do tratamento. E o *cluster* I.III contabilizou a média de 69.781 hab. e 50 municípios; ele teve a maior taxa de óbito no estrato I, bem como maiores valores de Gini, densidade domiciliar e ICSAP (Tabela 3).

O grupo II.I congregou, em média, 174.217 hab. e 801 municípios; no estrato II, ele registrou a maior média de cura. O agrupamento II.II foi composto por uma média de 182.628 hab. e 645 municípios; ele revelou maiores taxas de óbito e interrupção, assim como maiores valores de Gini, densidade domiciliar e ICSAP. E o cenário II.III teve média de 180.991 hab. e 1.247 municípios; ele apresentou os maiores percentuais de trabalhadores com ensino fundamental e cobertura de saúde suplementar, bem como maior expectativa de estudo (Tabela 3).

O *cluster* III.I contou com população média de 688.886 hab. e 688 municípios; ele teve as maiores taxas de cura e óbito no estrato III, bem como os valores mais altos de Gini, densidade domiciliar e ICSAP. O cenário III.II contabilizou uma média de 1.272.180 hab. e 812 municípios; ele registrou maiores valores de densidade domiciliar, expectativa de estudo e cobertura de saúde suplementar. E o grupo III.III teve, em média, 1.102.696 hab. e 747 municípios; ele apresentou maior taxa média de interrupção do tratamento (Tabela 3).

A região Norte possuiu maior representação do grupo I.I, além de outros grupos do estrato I. A região Centro-Oeste pareceu concentrar mais grupos do estrato I, mas com algumas incursões do estrato II. A região Sul, especialmente no Rio Grande do Sul e Paraná, teve maior predominância de grupos do estrato III, como III.I e III.III. A região Nordeste mostrou maior diversidade entre os estratos II e III, com algumas áreas do estrato I, dependendo do estado. E a região Sudeste apresentou uma combinação entre os estratos II e III, com menor participação do estrato I (Figura).

**Figura:** Distribuição espacial dos agrupamentos das regiões geográficas imediatas, segundo os *clusters* provenientes do método *k*-*means* por estrato populacional dessas regiões, Brasil, 2015–2021.

## DISCUSSÃO

Neste estudo, realizou-se a descrição das tendências dos resultados do tratamento da tuberculose em pessoas com HIV no Brasil. Embora as taxas de cura tenham permanecido predominantemente estáveis ao longo do período, tendências temporais crescentes para interrupção e óbito foram identificadas em alguns estados. Outrossim, padrões de desigualdade no agrupamento das regiões imediatas, com base em indicadores socioeconômicos e programáticos relacionados a esses desfechos, foram revelados no território nacional.

A estabilidade das taxas de cura, concomitante ao aumento de interrupções no seguimento e óbitos, indica uma provável redução na proporção de encerramentos não considerados nesta pesquisa, que se referem a preenchimentos operacionais do Sinan, como transferência e mudança de esquema. Essa diminuição pode refletir melhorias na classificação e no registro dos dados, além de alterações no perfil epidemiológico da população. Esse aspecto é crucial para compreender as dinâmicas dos encerramentos e pode ser detalhado em investigações futuras.

Estados que experienciaram aumentos na interrupção e no óbito podem se beneficiar de estratégias singularizadas de cuidado. No Brasil, recomenda-se o emprego de terapia antirretroviral para todas as pessoas com HIV como estratégia prioritária para melhorar os resultados do tratamento e, consequentemente, reduzir os agravos associados. Além disso, o uso concomitante da terapia antirretroviral durante o tratamento da tuberculose tem demonstrado eficácia na redução da mortalidade e na melhoria dos desfechos clínicos em pessoas com HIV^17^.

Nacionalmente, o estudo de Resende et al. demonstrou que a redução da carga viral do HIV e o uso de terapia antirretroviral foram os principais impulsionadores da diminuição da carga de tuberculose na população^18^. Nessa vertente, a implementação adequada dos medicamentos antirretrovirais pode ser crucial para a sobrevida de pessoas com coinfecção pela tuberculose, restaurando a função imunológica e prevenindo as formas mais graves da doença^19^. Não obstante, apenas 64,2% de todos os casos de coinfecção realizaram a terapia antirretroviral no Brasil em 2020^6^.

Os achados relacionaram a incidência de resultados desfavoráveis do tratamento da tuberculose a desigualdades socioeconômicas; o índice de Gini aumentou em 19% (IC95% 8% a 32%) a interrupção do tratamento. Na América Latina, uma revisão de escopo apontou que os principais fatores condicionantes para as iniquidades no acesso aos serviços de saúde são renda, escolaridade, transporte e moradia^20^. Isso sublinha as possíveis implicações das condições sociais na realização do tratamento da tuberculose nas regiões brasileiras.

Paradoxalmente, locais com altas taxas de ICSAP, isto é, com baixo desempenho da atenção primária, apresentaram menores percentuais de interrupção do tratamento e óbito por tuberculose nas pessoas com HIV. Infere-se que as hospitalizações podem funcionar como um ponto prioritário de cuidado em contextos de debilidade da atenção primária. No Brasil, a cobertura média da atenção básica em saúde figuraem 80%, com disparidades entre as regiões geográficas, sinalizando entraves no que se refere à expansão do acesso aos cuidados básicos^21^.

Embora a recomendação do tratamento da tuberculose em pessoas com HIV se paute no compartilhamento com os serviços de assistência especializada^17^, a atenção primária desempenha um papel crucial no estabelecimento de vínculo e na adesão às terapias medicamentosas^22^. Dessa forma, as internações por condições sensíveis, incluindo a tuberculose, podem ser entendidas como um mecanismo compensatório da rede de atenção – em especial nos casos de HIV, cuja descentralização do nível secundário ainda é incipiente.

Regiões com maior percentual de pessoas ocupadas com ensino fundamental tiveram altos percentuais de interrupção, ao passo que aquelas com maior expectativa de anos de estudo tiveram melhor desempenho. Em uma coorte chilena, verificou-se que pessoas com graduação tiveram menor probabilidade de óbito por coinfecção^23^. Nesse sentido, frisa-se que a garantia de acesso à educação nas regiões imediatas pode atuar como uma abordagem complementar na redução das disparidades imbricadas ao acesso e à vinculação ao tratamento da tuberculose no país.

A ocorrência de desfechos negativos também foi elevada em locais com maior densidade domiciliar (superior a duas pessoas por dormitório). Esse indicador mensura de forma indireta o perfil socioeconômico da população, sugerindo uma elevada vulnerabilidade familiar^24^. Domicílios densamente ocupados estão associados a questões de pobreza, que está ligada à má nutrição, a comorbidades e a condições precárias de vida; esses fatores facilitam a transmissão da tuberculose e agravam o quadro clínico de pessoas com coinfecção pelo HIV^25^.

O maior percentual de cobertura de saúde suplementar aumentou em 62% (IC95% 50% a 75%) a probabilidade de óbito por tuberculose em pessoas com HIV. A fragmentação entre os sistemas público e privado pode resultar em lacunas no acompanhamento de pessoas com coinfecção. Complementarmente, a saúde suplementar geralmente predomina em regiões maiores e mais bem desenvolvidas^26^. Nesse sentido, as desigualdades geradas nesses territórios podem dificultar o acesso ao tratamento, levando à ocorrência de piores resultados.

Diante do exposto, estratégias pautadas na vinculação ao cuidado são cruciais para oportunizar a melhoria do seguimento de pessoas em tratamento da dupla infecção. Deve-se considerar ferramentas capazes de estabelecer esse laço com os serviços, como o tratamento diretamente observado (TDO). Estudo em região da Malásia identificou que as pessoas com HIV que não receberam o TDO tiveram mais chances de descontinuar o tratamento ou evoluir a óbito por tuberculose (razão de chances – RC = 21,71; IC95% 5,36 a 87,94)^27^.

Como o contexto socioeconômico das regiões foi um importante aspecto atrelado ao tratamento, ações focalizadas em promover o acesso e a continuidade nos serviços de saúde em locais socioeconomicamente vulneráveis são indispensáveis. Especificamente no tratamento da tuberculose, o recebimento de incentivo financeiro em programas governamentais de transferência de renda já foi demonstrado como um fator negativamente associado à descontinuidade do tratamento em coorte do Sinan (RC = 0,64; IC95% 0,50 a 0,81)^28^.

Não obstante, no cenário brasileiro, a maior parte dos casos de tuberculose se concentra em municípios de grande porte com baixo desempenho em indicadores operacionais, incluindo testagem insuficiente para HIV e má implementação do TDO, além de baixa cobertura da atenção primária^29,30^. Nesse sentido, políticas estruturais devem garantir a saúde em um contexto mais abrangente, envolvendo medidas para mitigar as barreiras e iniquidades no acesso e na vinculação ao tratamento, haja vista as desigualdades sociais e de saúde aqui evidenciadas.

É preciso frisar que o uso de dados secundários constitui a maior limitação deste estudo, dada a possibilidade de subnotificação. Esses dados representam apenas as pessoas que acessaram o sistema; portanto, pode haver viés de sub-representatividade daquelas vivendo em áreas mais vulneráveis. No entanto, utilizou-se o relacionamento probabilístico entre quatro sistemas de informação (Sinan, SIM, Siclom ou Siscel) para aprimorar os dados estudados, sobretudo no que se refere à correção da subnotificação e à qualificação do desfecho.

Apesar da estabilidade nas taxas de cura da tuberculose em pessoas com HIV, viu-se aumento nas interrupções de seguimento e nos óbitos. As disparidades regionais na relação de indicadores socioeconômicos e programáticos com os desfechos da tuberculose sugerem iniquidades no acesso e na adesão ao tratamento da doença nos diferentes cenários do país. Requer-se o fortalecimento de políticas intersetoriais com foco no acesso e na adesão ao cuidado, com vistas a alcançar as metas de eliminação dessas infecções até 2030.

A contribuição deste estudo reside na descrição das tendências de aumento dos desfechos desfavoráveis do tratamento da tuberculose em pessoas com HIV. A identificação de agrupamentos apontou para a influência de desigualdades no acesso e na vinculação ao cuidado. Destarte, esta investigação pode colaborar com o desenvolvimento de políticas para qualificação da rede de atenção à saúde, bem como para mitigação das disparidades socioeconômicas. Investigações futuras se fazem necessárias para melhor compreender as associações observadas.
